# Carbon ion radiotherapy as definitive treatment in locally recurrent pancreatic cancer

**DOI:** 10.1007/s00066-021-01827-9

**Published:** 2021-08-05

**Authors:** Jakob Liermann, Edgar Ben-Josef, Mustafa Syed, Juergen Debus, Klaus Herfarth, Patrick Naumann

**Affiliations:** 1 Department of Radiation Oncology, Heidelberg University Hospital, Im Neuenheimer Feld 400, 69120 Heidelberg, Germany; 2 Heidelberg Institute of Radiation Oncology (HIRO), Im Neuenheimer Feld 400, 69120 Heidelberg, Germany; 3 National Center for Tumor Diseases (NCT), Im Neuenheimer Feld 460, 69120 Heidelberg, Germany; 4 Clinical Cooperation Unit Radiation Oncology, German Cancer Research Center (DKFZ), Im Neuenheimer Feld 280, 69120 Heidelberg, Germany; 5 Heidelberg Ion-Beam Therapy Center (HIT), Im Neuenheimer Feld 450, 69120 Heidelberg, Germany; 6 Department of Radiation Oncology, University of Pennsylvania, Philadelphia, USA; 7 German Cancer Consortium (DKTK), partner site Heidelberg, German Cancer Research Center (DKFZ), Im Neuenheimer Feld 280, 69120 Heidelberg, Germany

**Keywords:** Pancreatic cancer, Carbon ion radiotherapy, Particle therapy, Locally recurrent pancreatic cancer, Radiation oncology

## Abstract

**Purpose:**

Data on management of locally recurrent pancreatic cancer (LRPC) after primary resection are limited. Recently, surprisingly high overall survival rates were reported after irradiation with carbon ions. Here, we report on our clinical experience using carbon ion radiotherapy as definitive treatment in LRPC at the Heidelberg Ion-Beam Therapy Center (HIT).

**Methods:**

Between 2015 and 2019, we treated 13 patients with LRPC with carbon ions with a median total dose of 48 Gy (RBE) in 12 fractions using an active raster-scanning technique at a rotating gantry. No concomitant chemotherapy was administered. Overall survival, local control, and toxicity rates were evaluated 18 months after the last patient finished radiotherapy.

**Results:**

With a median follow-up time of 9.5 months, one patient is still alive (8%). Median OS was 12.7 months. Ten patients (77%) developed distant metastases. Additionally, one local recurrence (8%) and two regional tumor recurrences (15%) were observed. The estimated 1‑year local control and locoregional control rates were 87.5% and 75%, respectively. During radiotherapy, we registered one gastrointestinal bleeding CTCAE grade III (8%) due to gastritis. The bleeding was sufficiently managed with conservative therapy. No further higher-grade acute or late toxicities were observed.

**Conclusion:**

We demonstrate high local control rates in a rare cohort of LRPC patients treated with carbon ion radiotherapy. The observed median overall survival rate was not improved compared to historical in-house data using photon radiotherapy. This is likely due to a high rate of distant tumor progression, highlighting the necessity of additional chemotherapy.

## Background

Pancreatic cancer is one of the most aggressive tumors, with a 5-year overall survival (OS) rate of 5–10% [1]. The only curative treatment option is primary resection. Resection should always be performed if possible [2]. But even after surgery, approximately 80% of the patients die within 5 years [3, 4]. About a quarter of operated patients develop local tumor recurrence [5]. If the tumor burden remains restricted to the pancreas or to the operation bed, local treatment options should be considered. This is significant, as approximately 30% of all pancreatic cancer-related deaths are due to local tumor burden [6]. Data on management of locally recurrent pancreatic cancer (LRPC) are scarce. Re-resection is considered the best treatment option, as several reports demonstrated a postoperative median OS of 25–26 months [7, 8] and a pooled analysis showed a 5-year OS rate of 40.6% [9]. Conventional fractionated photon radiotherapy in LRPC has been investigated in several retrospective reviews, but efficacy remains poor [10, 11]. However, in inoperable cases, radiotherapy or chemoradiotherapy should be discussed.

Modern radiation techniques such as stereotactic body radiotherapy (SBRT) or particle therapy are characterized by a steeper dose gradient. This gradient results in an improved sparing of organs at risk (OARs), allowing delivery of a higher biological effective dose (BED) to the gross tumor volume (GTV). The efficacy of SBRT in LRPC was analyzed by Comito et al., who observed a median OS of 18 months after irradiating with 45 Gy in 6 fractions [12]. In 2018, Ryan et al. reported on 51 patients treated with SBRT with a total dose of 25–33 Gy in 5 fractions, mostly (59%) followed by maintenance chemotherapy [13]. The median OS was 16 months, although the study included patients receiving re-irradiation. For response evaluation of radiotherapy, highly precise imaging techniques should be used [14].

Particle therapy could be more effective than SBRT in the management of LRPC due to its biological and/or physical advantages over photon radiotherapy [15]. Mizumoto et al. observed a median OS of 26.1 months after radiotherapy with protons with a total dose of 67.5 Gy (RBE) in 19–25 fractions, mostly (83%) combined with chemotherapy [16]. Gastrointestinal bleeding was frequent (43.3%). One patient (3.3%) developed grade III bleeding. Another three patients (10%) developed grade III spinal fractures.

The only data available on the efficacy of carbon ion radiotherapy in LRPC were published in 2018. In this retrospective analysis, Kawashiro et al. demonstrated a median OS of 25.9 months after carbon ion radiotherapy with a total dose of 52.8–55.2 Gy (RBE) in 12 fractions, predominantly (57%) combined with chemotherapy [17]. Radiotherapy with carbon ions seemed safe, without any high-grade radiation-induced toxicity. These promising data explain the growing interest in the efficacy of particle therapy in pancreatic cancer, but no additional supporting results have been published.

In the present study, we analyzed the feasibility, safety, and efficacy of irradiation with carbon ions in LRPC patients at the Heidelberg Ion-Beam Therapy Center (HIT).

## Methods

### Patients

**Fig. 1:**
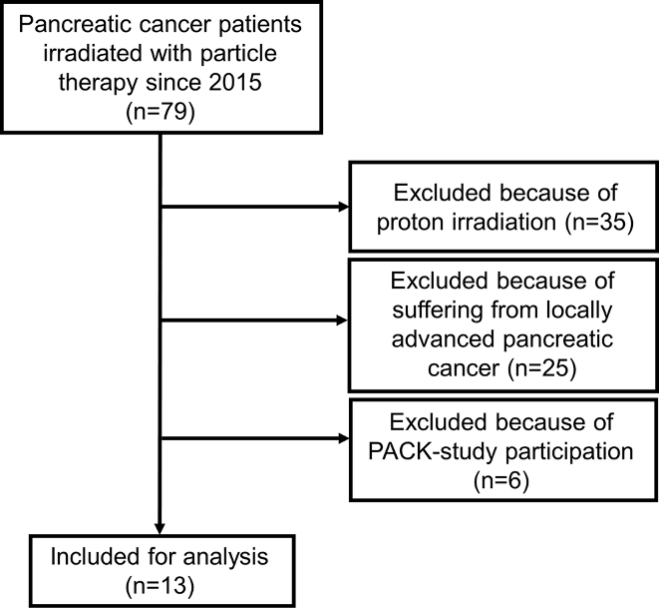
Consort diagram of the patient inclusion procedure

All patients suffering from LRPC after primary resection who were irradiated with carbon ions at our institution were included in this study. Clinical information was extracted from the charts. Patients participating in the ongoing PACK trial [18] were excluded from the analysis (Fig. 1). Tumor staging needed to be done by computed tomography of the chest and the abdomen within 3 months prior to irradiation. Patients with distant metastases were not deemed suitable for carbon ion radiotherapy, although one patient presented with a pre-radiotherapy American Joint Committee on Cancer (AJCC) stage IV due to a peritoneal tumor lesion that was removed during the initial Whipple procedure.

### Radiotherapy

For radiation planning, patients underwent contrast-enhanced four-dimensional computed tomography (4D-CT) with a slice thickness of 3 mm. Radiation planning was performed using the inverse treatment planning system Syngo RT Planning (Siemens, Erlangen, Germany). Patients were irradiated in free-breathing using an internal target volume (ITV) concept and should have fasted for 3 hours prior to irradiation. The underlying biological plan optimization includes the local effect model (LEM) I, developed at the GSI Helmholtzzentrum für Schwerionenforschung and at HIT [19, 20]. The α/β ratio for LEM I was mostly (92%) set at 5 Gy for the ITV and at 2 Gy for the organs at risk (OARs). A total dose of 48 Gy (RBE) was delivered in 12 fractions in all but one case. In the remaining case, 44 Gy (RBE) was delivered in 11 fractions. Forty-eight Gy (RBE) corresponds to an equivalent dose at 2 Gy (EQD2) of 61.7 Gy and to a BED of 86.4 Gy, assuming an α/β ratio of 5 Gy (α/β = 2 Gy: EQD2 = 72 Gy, BED = 144 Gy). Treatment planning and irradiation were mostly performed in supine position (92%) using two dorsal oblique radiation beams (92%). One patient was irradiated in prone position and another patient received irradiation using a single radiation beam.

The following dose constraints for the OARs were given but could not always be respected: less than 20% of the kidney volume should receive more than 24 Gy (RBE); maximum dose in the spinal cord was set at 36 Gy (RBE); in the upper gastrointestinal tract, no more than 43.2 Gy (RBE) should be delivered; the liver should be irradiated as low as reasonably achievable (ALARA).

### Target volume definition

The gross tumor volume (GTV) was defined as macroscopic tumor on imaging. The clinical target volume (CTV) accounting for microscopic tumor spread was individually adjusted. Two patients (15%) presented with regional lymphatic metastases that were included in the CTV. The ITV consisted of the CTV that was adjusted for respiratory movement. An expansion of the ITV by 5 mm and 7 mm in beam direction was used to generate the planning target volume (PTV). Radiotherapy was performed with an intensity-controlled raster-scanning system for beam application at a rotating gantry.

### Follow-up and response evaluation

Follow-up was defined from the first day of radiation until last clinical evaluation or death. Follow-up was performed every 3 months by contrast-enhanced CT scans and clinical evaluation, whenever this was available. Tumor response was evaluated according to the RECIST 1.1 criteria [21]. Local tumor recurrence was defined as tumor progression within the high-dose (> 90% of the prescribed dose) radiation area. Tumor recurrences outside this area were denominated as regional tumor recurrence, if restricted to the pancreas or the operation bed with its adjacent lymphatics. Any other tumor recurrence was defined as distant tumor recurrence.

The OS was defined as time from the start of radiotherapy until reported death due to any cause. Local control (LC) was defined from the start of radiotherapy until local tumor recurrence or last imaging available. Locoregional control (LRC) was defined from the start of radiotherapy until local or regional tumor recurrence or last imaging available. Freedom from distant metastasis (FFDM) was defined from the start of radiotherapy until first occurrence of distant metastasis or last imaging available. Progression-free survival (PFS) was defined as time from the start of radiotherapy until any tumor progression or death or last imaging available.

### Toxicity evaluation

Toxicity was defined according to the International Common Terminology Criteria for Adverse Events of the National Cancer Institute (NCI CTCAE), version 5. Acute toxicity included symptoms that occurred < 3 months after irradiation. Late toxicity was defined as symptoms that lasted for ≥ 3 months after radiotherapy.

### Statistics

OS, LC, LRC, FFDM, and PFS were analyzed using the Kaplan–Meier method. Statistics and figures were performed with SPSS Statistics, version 27 (International Business Machines Corporation: IBM, Armonk, NY, USA).

## Results

### Patient and treatment characteristics

**Fig. 2:**
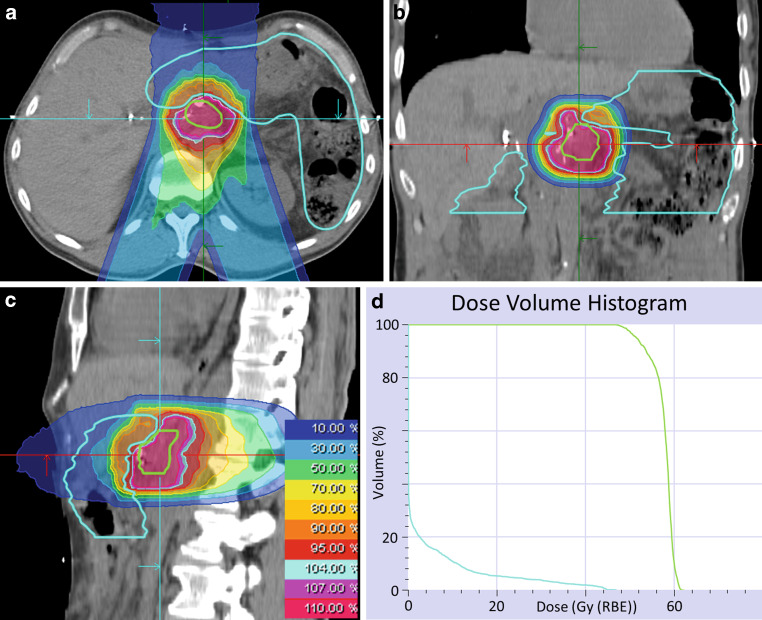
Radiation plan of a 59-year-old patient suffering from locally recurrent pancreatic cancer demonstrating a steep dose gradient of the performed irradiation. The patient was irradiated in supine position using two oblique posterior beams to avoid gastrointestinal toxicity. Axial (**a**), coronal (**b**), and sagittal (**c**) computed tomography (CT) slices and isodose lines are shown. The gross tumor volume (GTV) is delineated in *green*, the gastrointestinal tract is contoured in *light blue*. Isodoses represent a forward-calculation using an α/β ratio of 2 Gy in the local effect model (LEM) I. Therefore, the GTV seems to be overdosed. In the actually irradiated plan, an α/β ratio of 5 Gy in LEM I was used for the tumor tissue. **d **Dose–volume histogram of the radiation plan demonstrating adequate coverage of the GTV (*green*) while avoiding overdosage in the gastrointestinal tract (*light blue*)

Between May 2015 and February 2019, 13 LRPC patients were irradiated with carbon ions at HIT. A median total dose of 48 Gy (RBE) was delivered in 12 fractions using an active raster-scanning technique at a rotating gantry. No concomitant chemotherapy was administered. Patient and treatment characteristics are shown in Tables 1 and 2. A representative radiation plan is shown in Fig. 2.

**Table 1:** Patient characteristics

	*n*	(%)
Number of patients	13	(100)
*Sex*		
Male	5	(38)
Female	8	(62)
*Age at radiotherapy (median in years, range)*	70 (48–77)	–
*Localization of initial pancreatic cancer*		
Pancreatic head	9	(69)
Pancreatic body	3	(23)
Pancreatic tail	1	(8)
*Initial AJCC stage*		
IA	1	(8)
IIA	5	(38)
IIB	3	(23)
III	3	(23)
IV	1	(8)
*Preoperative chemotherapy*		
FOLFIRINOX	4	(31)
None	9	(69)
*Surgery*		
Whipple procedure	8	(62)
Total pancreatectomy	3	(23)
Distal pancreatectomy	2	(15)
*Department of Surgery*		
Heidelberg University Hospital	9	(69)
Other	4	(31)
*Resection status*		
RX	1	(8)
R1	8	(62)
R0	4	(30)
*Histology*		
Ductal adenocarcinoma	13	(100)
*Grading*		
G1	1	(8)
G2	6	(46)
G3	3	(23)
Unknown	3	(23)
*Postoperative chemotherapy*		
FOLFIRINOX	4	(31)
Gemcitabine-based chemotherapy	6	(46)
Unknown chemotherapy	1	(8)
None	2	(15)

*AJCC* American Joint Committee on Cancer; *FOLFIRINOX* Chemotherapy regimen consisting of folinic acid, fluorouracil, irinotecan, and oxaliplatin

**Table 2:** Treatment characteristics

	*n*	(%)
Radiotherapy		
*Time in months: resection to local tumor recurrence (median, range)*	14 (4–41)	–
*Time in months: resection to radiotherapy (median, range)*	19 (6–51)	–
Pre-radiotherapy AJCC* stage		
III	12	(92)
IV	1	(8)
*Radiation technique*		
Carbon ions, active raster-scanning	13	(100)
*Prescribed dose*		
48 Gy (RBE) in 12 fractions	12	(92)
44 Gy (RBE) in 12 fractions	1	(8)
*Concurrent chemotherapy*		
None	13	(100)
*Patient position*		
Supine	12	(92)
Prone	1	(8)
*Volume in ccm (median, range; mean, standard deviation)*		–
GTV (gross tumor volume)	21.5 (7.3–340.0); 46.8 (101.0)	
CTV (clinical target volume)	66.2 (25.6–569.3); 105.8 (156.0)	
ITV (internal target volume)	85.4 (44.8–679.6); 140.4 (183.5)	
PTV (planning target volume)	165.0 (91.5–1007.1); 238.9 (263.3)	
*Number of radiation beams*		
2	12	(92)
1	1	(8)
*Postradiation chemotherapy*		
FOLFIRINOX	3	(23)
Gemcitabine-based chemotherapy	3	(23)
Unknown chemotherapy	1	(8)
None	6	(46)

*AJCC* American Joint Committee on Cancer; *FOLFIRINOX* Chemotherapy regimen consisting of folinic acid, fluorouracil, irinotecan, and oxaliplatin

### Follow-up

The analysis was performed 18 months after the last patient finished radiotherapy. Median follow-up time was 9.5 months and, in the alive patient, 33.8 months. Median time interval from the start of radiotherapy until the last available imaging was 8.4 months.

### Overall survival

**Fig. 3:**
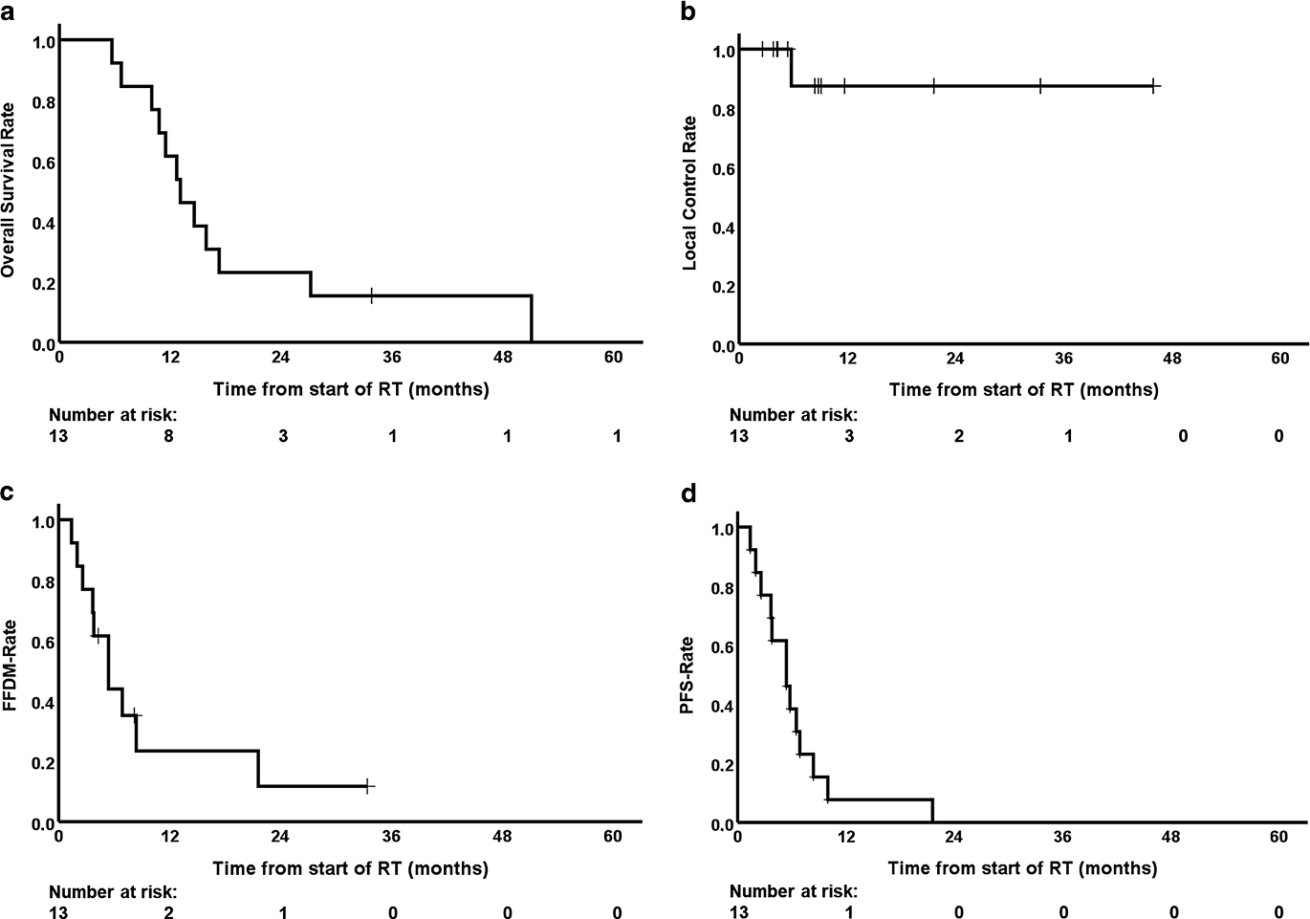
Based on Kaplan–Meier estimates, **a** overall survival (OS), **b** local control (LC), **c** freedom from distant metastasis (*FFDM*), and **d** progression-free survival (*PFS*) of 13 patients. All patients were suffering from locally recurrent pancreatic cancer (LRPC) and underwent carbon ion radiotherapy (*RT*) at the Heidelberg Ion-Beam Therapy Center (HIT)

Median OS was 12.7 months with a 95% confidence interval (CI) of 7.4–18.0 (Fig. 3a). At the time of analysis, one patient was still alive. The 1‑year and 2‑year OS rates were 58.3% and 25%, respectively. Median OS after initial resection was 45.2 months (95% CI 18.8–71.6).

### Local control and locoregional control

**Fig. 4:**
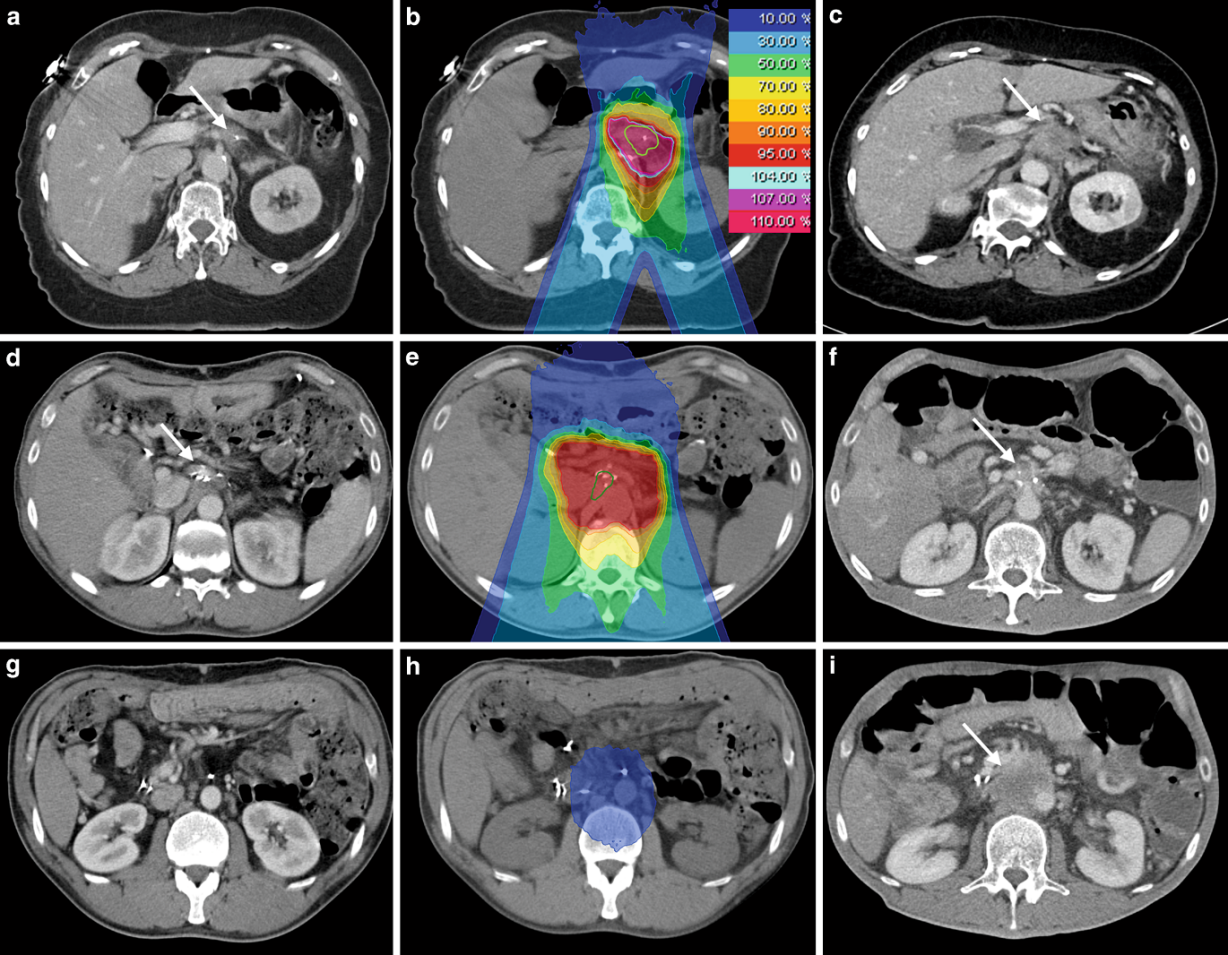
**a–c** Baseline imaging (**a**), radiation plan (**b**), and follow-up imaging (**c**) of a locally recurrent pancreatic cancer (LRPC) patient showing local tumor recurrence (*white arrows*) after carbon ion radiotherapy. **d–f** Baseline imaging (**d**), radiation plan (**e**), and follow-up imaging (**f**) of another LRPC patient showing stable local disease (*white arrows*) after radiotherapy. **g–i** Corresponding images of the same patient as in **d–f** at a more distal location, showing regional tumor recurrence (*white arrow*) in follow-up imaging (**i**). The tumor recurrence occurred in the low-dose area (10% isodose line) of the performed radiation (**h**) and was therefore defined as regional tumor recurrence

Local tumor recurrence was observed in one patient 6 months after carbon ion radiotherapy (Fig. 4a–c). This patient died 4.5 months later. The estimated 1‑year LC rate was 87.5% (Fig. 3b). Another two patients presented with regional tumor recurrences outside of the high-dose radiation area. These regional tumor recurrences occurred 7 months and 3.5 years after carbon ion radiotherapy. The corresponding estimated 1‑year locoregional control was 75%. One representative regional tumor recurrence is shown in Fig. 4d–f and 4g–i.

### Freedom from distant metastasis and progression-free survival

Distant metastasis developed in 10 patients. The median time interval between radiotherapy and occurrence of distant metastasis was 5.4 months (95% CI 2.9–7.9). After 6 and 12 months, an estimated 66.0% and 88.3% of the patients presented with distant tumor recurrence, respectively (Fig. 3c). With a median PFS of 5.4 months (95% CI 3.1–7.7), an estimated 8% of the patients were free of any tumor progression after 1 year (Fig. 3d).

### Toxicity

One patient developed a gastric hemorrhage CTCAE grade III during radiotherapy. This patient initially presented with a large LRPC with regional lymph node metastases. Therefore, the corresponding PTV was the largest of the cohort (1007 ccm, Table 2). The patient presented with bloody stools after having been irradiated with a total dose of 36 Gy (RBE). The maximum radiation dose (Dmax) in the stomach was at 35.6 Gy (RBE) at that timepoint, the Dmax in the small intestine was at 39.4 Gy (RBE). The patient required transfusion and was temporarily monitored in the intensive care unit of our institution (< 24 h). Endoscopic examination did not reveal the exact source of the bleeding, but radiotherapy-induced gastritis was assumed to be the reason for the hemorrhage. The patient recovered fast with conservative therapy. After a break of three radiation fractions, he resumed and completed radiotherapy without any further bleeding. Nevertheless, the radiation plan was modified, and it was decided to reduce the total dose to 44 Gy (RBE) to lower the risk of gastrointestinal toxicity. During follow-up there was no further suspicion of gastrointestinal ulcer. Toxicity rates are shown in Table 3.

**Table 3:** Toxicity rates

Symptoms (NCI CTCAE grades)	Before RT *n* (%)	Acute toxicity *n* (%)	Late toxicity *n* (%)
*Abdominal pain*			
I	4 (31)	1 (8)	3 (23)
II	1	3 (23)	1 (8)
*Gastric hemorrhage*			
III	0	1 (8)	0
*Gastritis*			
II	0	1 (8)	0
*Diarrhea*			
I	4 (31)	3 (23)	1 (8)
II	1 (8)	0	0
*Ascites*			
I	1 (8)	1 (8)	1 (8)
II	0	1 (8)	0
*Nausea*			
I	5 (38)	3 (23)	1 (8)
II	0	2 (15)	0
*Dermatitis*			
I	0	1 (8)	0
*Fatigue*			
I	2 (15)	2 (15)	2 (15)
II	0	2 (15)	0
No complaints	3 (23)	5 (38)	3 (23)

*RT* radiotherapy, *NCI CTCAE* Common Terminology Criteria for Adverse Events of the National Cancer Institute

## Discussion

In the present study, we analyzed carbon ion radiotherapy in locally recurrent pancreatic cancer for the first time in Europe. Furthermore, the abovementioned retrospective study of Kawashiro et al. provides the only published data concerning carbon ion radiotherapy in LRPC worldwide [17]. In our study, we could demonstrate encouraging local tumor control rates with a 1-year LC rate of 87.5%. This finding could be biased by the observed low OS, since patients might not have reached the endpoint of local tumor recurrence. Nevertheless, the observed LC is consistent with the hypothesis of improved efficacy when irradiating with higher RBE and BED. Furthermore, the results are comparable to local control rates with carbon ion radiotherapy in unresectable locally advanced pancreatic cancer published by Shinoto et al. [22].

We observed high-grade toxicity in only one patient, in line with the low toxicity rates published by Kawashiro et al. Taking account of the fact that the patient who developed grade III gastric hemorrhage had presented with the largest tumor volume of the cohort, one could state that, at least for limited tumor volumes, the dose schedule used is safe and feasible.

Compared to historical data of photon radiotherapy in LRPC, the observed median OS was not improved. In an in-house study with 41 patients, Habermehl et al. reported a median overall survival of 16.1 months after photon radiotherapy with a median total dose of 48.4 Gy in 28 fractions, mostly (90%) combined with chemotherapy with gemcitabine 300 mg/m^2^ body surface [10]. Nakamura et al. observed similar results irradiating with photons to a median total dose of 54 Gy in 30 fractions, predominantly (60%) combined with chemotherapy [11]. In the present study, the median OS of 12.7 months seems lower than photon-based results. There is also a discrepancy between our findings and the observed median OS of 25.9 months after carbon ion radiotherapy in LRPC described by Kawashiro et al. [17] The following could explain these differences.

First, the underlying patient cohorts differed widely. Compared to the present study, the investigated patients were younger in the analysis of Kawashiro et al. (70 years vs. 61 years). Also, men were underrepresented in our study (38%). Only 57% of the patients of Kawashiro et al. suffered from pancreatic head cancer. In our cohort, this subgroup was higher (69%). Irradiating tumors in the area of the pancreatic head, gastrointestinal toxicity risk is elevated due to the proximity to the duodenum. Consequently, the radiation dose must be restricted to lower dose concepts to avoid toxicity. Also, the rate of margin-free (R0) tumor resections was much higher in Kawashiro et al.’s patient cohort (70% compared to 30% in the presented study). This is significant, as R0 resection correlates strongly with improved survival rates in pancreatic cancer [23, 24]. Furthermore, the median PTV of the study of Kawashiro et al. was slightly smaller than the one presented in the current analysis (162.6 ccm, range: 47.3–347.8 ccm vs. 165.0 ccm; range: 91.5–1007.1 ccm). Altogether, the analysis of Kawashiro et al. seems to be based on healthier patients with lower tumor burden compared to the present study.

Secondly, the treatment regimen was different. Kawashiro et al. irradiated with a total dose of 52.8–55.2 Gy (RBE) in 12 fractions. This dose concept seems higher than the one in the present series, i.e., 48 Gy (RBE) in 12 fractions. However, heavy ion doses cannot be compared nominally, as different RBE calculation models and different beam applications are used in Japan and at HIT [25]. In our study, we decided not to irradiate with doses higher than 48 Gy (RBE) to avoid gastrointestinal toxicity. The observed CTCAE grade III toxicity suggests that this LEM I-based dose schedule is near the maximum tolerable irradiation dose in our facility.

Most of the patients of the study of Kawashiro et al. were treated with concomitant chemotherapy, whereas patients in the present cohort did not receive concurrent chemotherapy. There are higher rates of distant tumor progression in the present study compared to the patient cohort of Kawashiro et al. (77% vs. 63%), and distant progression occurred early. Therefore, combining carbon ion radiotherapy with chemotherapy could be important for improving oncological outcome.

The patient cohort of 13 patients is too small to draw definitive conclusions. Nevertheless, it is worth describing this rare patient cohort for which few data on management exist. We believe that some of the lessons gleaned from this experience can be applied to other settings in which carbon ion radiotherapy is used in the abdomen. Given the limited number of carbon ion radiotherapy facilities worldwide, very few reports on clinical outcomes have been published. There is growing interest in particle radiotherapy in pancreatic cancer, since promising results have recently been reported in Japan [17, 22, 26–28]. Currently, the ongoing phase II PACK trial is prospectively investigating carbon ion radiotherapy in non-metastasized pancreatic cancer and will deepen knowledge in this promising field of research [18].

## Conclusion

In the present study, we could demonstrate excellent local tumor control rates and low toxicity rates after carbon ion radiotherapy in LRPC. However, we could not confirm improvement of OS rates. Based on our findings, systemic treatment should be considered concomitantly and after carbon ion radiotherapy of locally recurrent pancreatic cancer patients due to the observed high rate of distant metastases.
